# On the Question of Zwitterionic Intermediates in the [3 + 2] Cycloaddition Reactions between C-arylnitrones and Perfluoro 2-Methylpent-2-ene

**DOI:** 10.3390/molecules26237147

**Published:** 2021-11-25

**Authors:** Katarzyna Mitka, Katarzyna Fela, Aleksandra Olszewska, Radomir Jasiński

**Affiliations:** Department of Organic Chemistry and Technology, Cracow University of Technology, Warszawska 24, 31-155 Cracow, Poland; katarzyna.mitka@pk.edu.pl (K.M.); katarzyna.fela@pk.edu.pl (K.F.); aleksandra.o1@o2.pl (A.O.)

**Keywords:** [3 + 2] cycloaddition, nitrones, DFT study, molecular mechanism

## Abstract

The molecular mechanism of the [3 + 2] cycloaddition reaction between C-arylnitrones and perfluoro 2-methylpent-2-ene was explored on the basis of DFT calculations. It was found that despite the polar nature of the intermolecular interactions, as well as the presence of fluorine atoms near the reaction centers, all reactions considered cycloaddition proceed via a one-step mechanism. All attempts for the localization of zwitterionic intermediates on the reaction paths were not successful. Similar results were obtained regardless of the level of theory applied.

## 1. Introduction

The presence of fluorine atoms in organic molecules stimulates some valuable physico-chemical properties from a practical point of view. Formally, the C-F single bond is only 0.27 Å longer than the respective C-H bond [1]. However, the energetical stability of both mentioned bonds is substantially different [2]. On the other hand, the high electronegativity of fluorine within the classical Pauling scale determines the polar nature of C-F bonding interactions. The specific properties of fluoroorganic compounds, such as pKa, steric effects, lipophilicity, inductive effects, hydrogen bonding, and isosterism, provide a wide range of applications in medicinal chemistry [3]. In particular, the C-F- moiety is a key element in thymidylate synthase inhibitors (such as galocitabine, emitefur, ftorafur, and fluorocytosine), topoisomerase inhibitors (such as clofarabine and fluodarabine), microtubule-stabilizing agents (such as paclitaxel), multi-drug efflux protein resistance (raloxifene and zosuguidar), protein kinase inhibitors (gefitinib), anti-androgens (nilitamide, fluramide), non-steroidal anti-inflammatory drugs (colecoxib and diflunisal) [4], and antimicrobial agents [5], as well as drugs devoted to Alzheimer’s disease [6]. Some fluoroorganic compounds play an effective xenobiotic function in peptide backbone modification [7]. Fluorine is also emerging as one of the evidently prominent atoms in medical diagnostics using positron emission tomography (PET) due to the favorable half-life of the ^18^F isotope (109.8 min) when compared to ^11^C (20.4 min) and ^124^I (4.2 days). This is especially important for the preparation of drugs dedicated to the central nervous system [8,9,10].

**Scheme 1:**
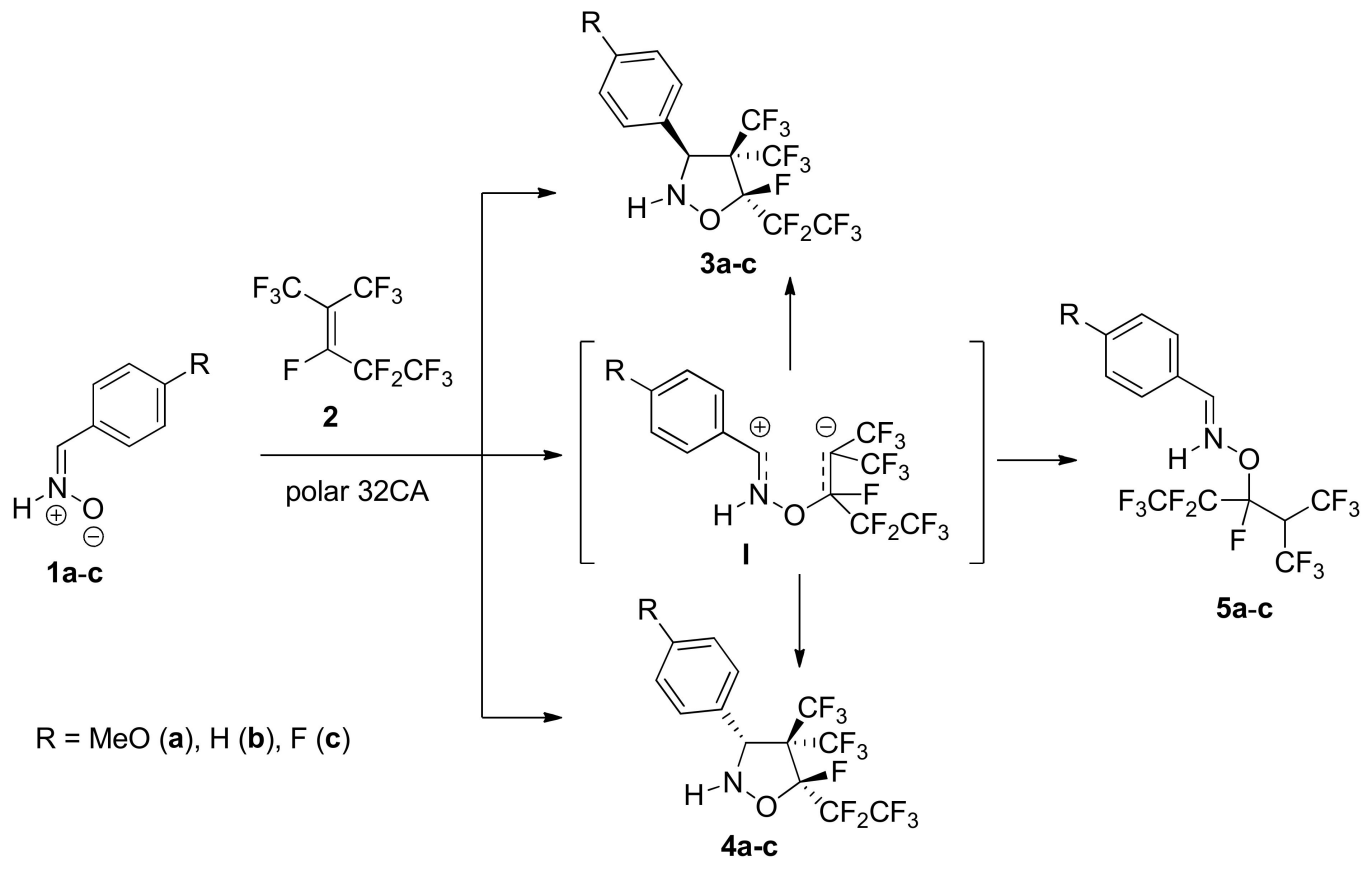
General course and proposed molecular mechanism for 32CAs between C-arylnitrones (**1a**–**c**) and perfluoro 2-methylpent-2-ene (**2**).

Some years ago, Lee and coworkers [11] described the interesting case of a [3 + 2] cycloaddition reaction (32CA) between rare examples of mono-C-substituted nitrones (**1a**–**c**) and perfluoro 2-methylpent-2-ene (**2**). This reaction proceeded under mild conditions and in a full regioselective manner, leading to stereoisomeric isoxazolidines determined by different 3,5-cis- and 3,5-trans configurations (**3a**–**c** and **4a**–**c**). However, in the reaction mixtures, acyclic adducts **5a**–**c** were also detected. The presence of this type of adduct suggests that the cycloaddition process might proceed via a zwitterionic intermediate (Scheme 1). This assumption is justified by the polar nature of the interaction between the reaction components [12,13,14]. Besides this, it is generally known that the fluoroalkyl substituents stabilize ionic centers within zwitterions structures [15,16,17]. Consequently, the molecular mechanism of the mentioned cycloadditions requires a full exploration.

Even 30–40 years ago, it was believed that [3 + 2] cycloaddition reactions could only proceed according to a one-step “concerted” mechanism [18,19]. Currently, however, numerous cases of such reactions are known to proceed through intermediates of zwitterionic or diradical nature. The zwitterionic mechanism is especially favored by polar interactions between addends, different shielding of reaction centers, polar reaction environments, and the presence of substituents stabilizing ionic centers [20,21,22].

**Scheme 2:**
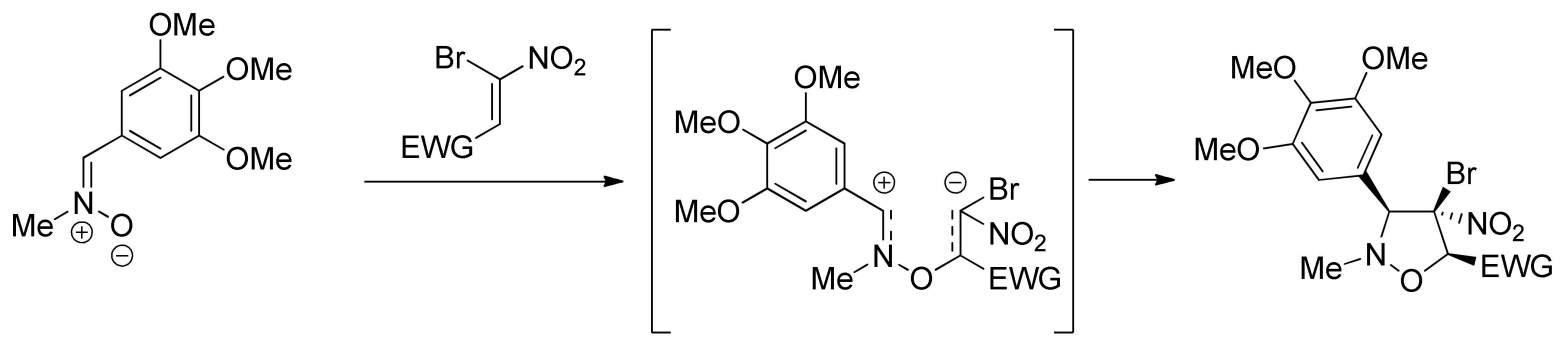
The stepwise, zwitterionic mechanism of 32CAs of *Z*-C-(3,4,5-trimethoxyphenyl-N-methylnitrone and *Z*-2-EWG-1-bromo-1-nitroethenes.

It should be underlined that, in recent literature, some examples of stepwise, zwitterionic mechanisms of 32CAs involving nitrones have been described. For example, zwitterionic intermediates were detected as the key stage in the 32CA reaction between *Z*-C-(3,4,5-trimethoxyphenyl-N-methylnitrone and *Z*-2-EWG-1-bromo-1-nitroethenes (Scheme 2) [23].

**Scheme 3:**
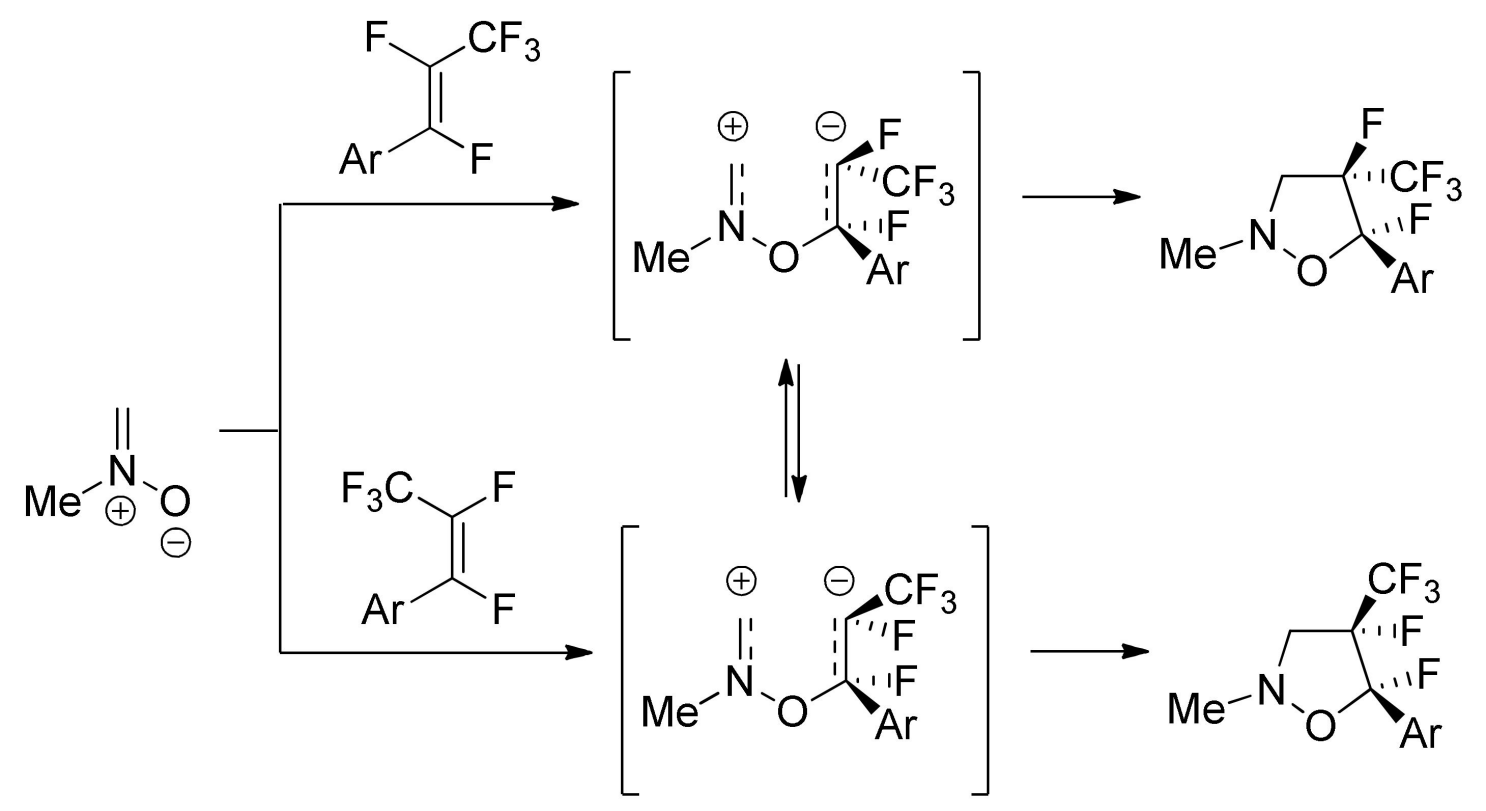
The stepwise, zwitterionic mechanism of 32CAs of *N*-methylnitrone and 1,1,1,2,3-pentafluoro-3-arylethenes.

A stepwise mechanism was also proposed for the 32CA between *N*-methylnitrone and 1,1,1,2,3-pentafluoro-3-arylethenes based on a stereochemical criterion (Scheme 3) [24].

Interesting examples of stepwise, zwitterionic mechanisms were also described regarding 32CAs involving the participation of other three-atom-components (TACs), such as nitronic esters [25], tiocarbonyl ylides [26,27], azzomethine ylides [28], diazocompounds [29,30], nitrile N-oxides [31,32], and azides [33].

## 2. Results and Discussion

In the present study, the nature of the intermolecular interactions between the cycloaddition components was analyzed first. This analysis was performed based on CDFT reactivity indices (Table 1) [34]. A similar approach was recently used to explain the course of many different types of cycloaddition processes [35,36,37,38]. It was found that nitrones **1** exhibited a moderately electrophilic nature. At the same time, all of these nitrones were characterized by high values of global nucleophilicity (N > 3.4 eV). On the other hand, perfluoro 2-methylpent-2-ene **2** was classified as a markedly strong electrophile (ω > 2 eV). Thus, the global interaction in the considered 32CA processes would clearly have a polar nature. This excludes the possibility of a reaction course via biradical intermediates, but does not exclude the possible existence of zwitterionic intermediates.

It is well-known that the regioselectivity in polar processes can be explained on the basis of local reactivity indices. In the case of the analyzed nitrones, the most nucleophilic reaction center was the oxygen atom of the >C=N(O)- moiety (local nucleophilicity in the range of 2.05–2.24 eV; see Table 1). On the other hand, the most electrophilic reaction center in the alkene structure was the C2 carbon atom (ω _C2_ = 1.12 eV). Local interactions between the mentioned centers determined the formation of cycloadducts with the CF_3_ at the 4- position of the heterocyclic ring. This interpretation agrees with the regioselectivity experimentally observed [11].

**Figure 1:**
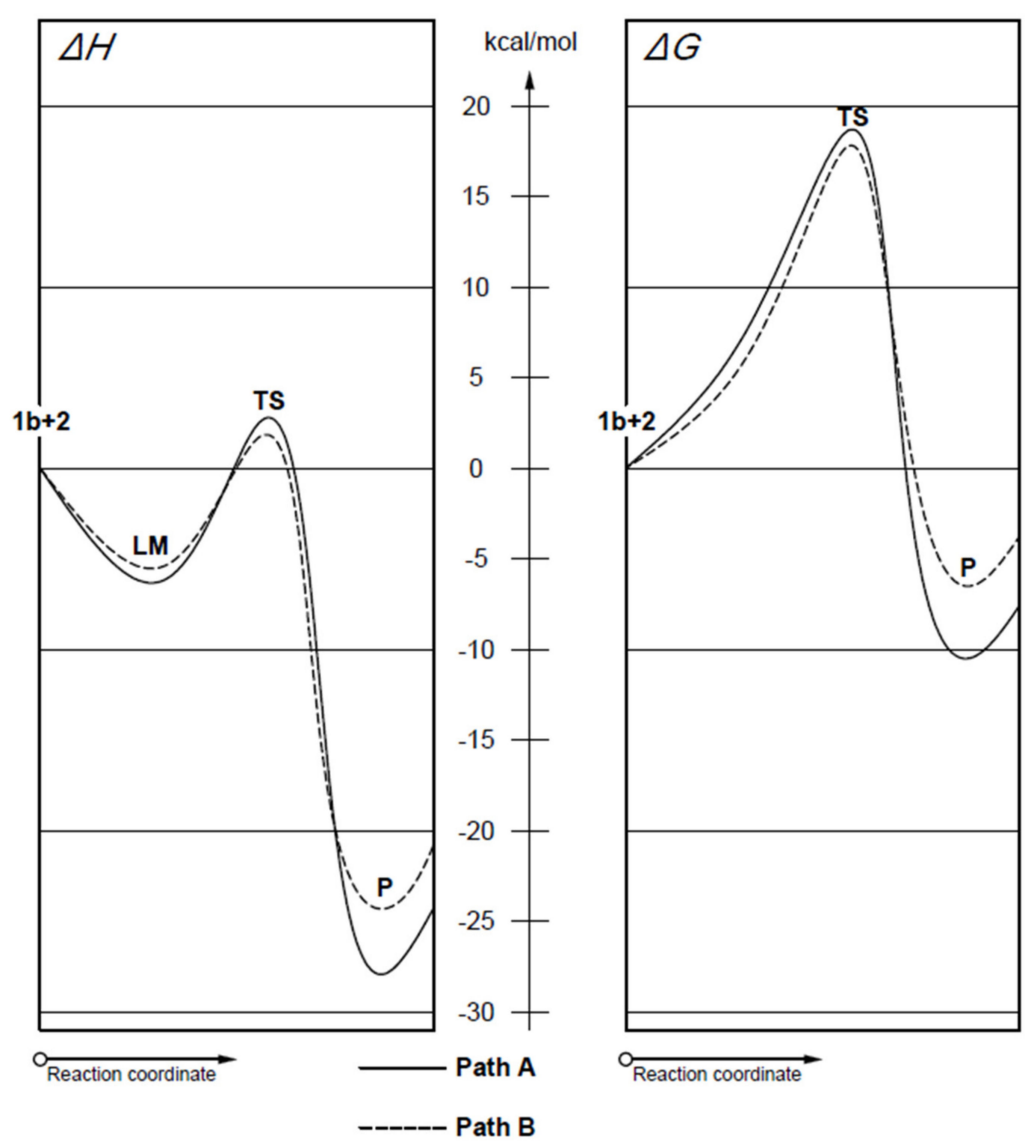
B3LYP/6-31G(d) gas phase energy profiles for the 32CAs of nitrone **1b** and perfluoro 2-methylpent-2-ene **2**.

The analysis of CDFT indices sheds light on the nature of the intermolecular interactions in the title reactions. Unfortunately, this approach cannot give any information regarding critical structures along the reaction path. Therefore, in the second step, the experimentally observed reaction channels were fully explored. First, the model reaction involving nitrone **1b** was studied in the gas phase. In both considered reaction channels, two critical points between valleys of substrates and products were localized (Figure 1).

In the gas phase, the interactions between addends at the initial step led to a minimum associated with the existence of a pre-reaction molecular complex (**MC**). This was accompanied by a reduction in the enthalpy of the reaction system by about a few kcal/mol (Table 2). The significant increase in the entropy ensured, however, that the Gibbs free energies of the **MC**s formation were positive. This excludes the possibility of the **MC**s′ existence as stable intermediates. Within the **MC**s, the substructures derived from the addends approached each other, providing favorable electrostatic interactions to the cycloaddition process, but not enough to allow the formation of the new single bonds (Table 3). In particular, for both **MCA** and **MCB,** the C3–C4 distances were 3.82 Å, whereas the C5-O1 distances were about 2.7 Å. Thus, the interatomic distances were beyond the typical range for the corresponding distances at transition states [36,37,38,39,40]. It should be underlined that these **MC**s did not exhibit the nature of electron density transfer complexes [41]. This was evident from the GEDT values (0.00e, Table 3).

**Figure 2:**
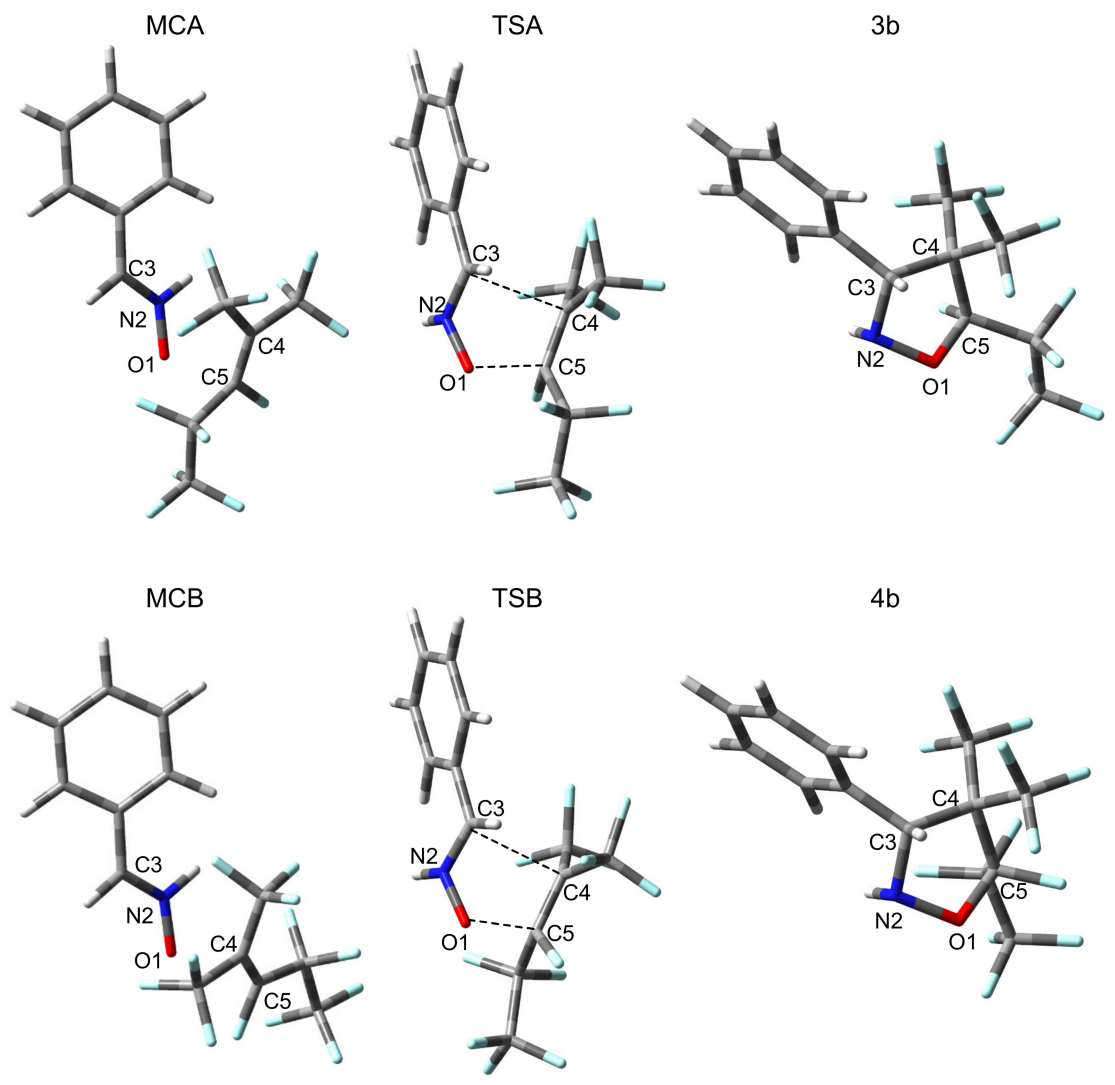
Views of the key structures involved in the 32CAs of nitrone **1b** and perfluoro 2-methylpent-2-ene **2** obtained in gas phase from B3LYP/6-31G(d) calculations.

The further conversion of **MC**s within both considered paths directly led to the transition states (**TS**). This was accompanied by an increase in the system enthalpy to 2.8 kcal/mol and 1.9 kcal/mol for paths **A** and **B**, respectively. Therefore, both reaction channels should be considered allowed from a kinetics point of view (Figure 1, Table 2). In **TS**s, the reaction centers exhibited the orientation that allowed the formation of new single bonds (Figure 2). In particular, the C3-C4 distances reached values of 2.511 Å and 2.532 Å at **TSA** and **TSB,** respectively. Likewise, the C5-O1 distances were 1.816 Å and 1.764 Å at **TSA** and **TSB,** respectively. This observation allows the conclusion that the reaction course is determined by the nucleophilic attack of the oxygen atom from the >C=N(O)- molecular segment of the nitrone on the C2 center of the alkene (Figure 2). This conclusion perfectly agrees with the analysis of the local reactivities of the addends. Both **TS**s exhibited a polar nature, which was confirmed by high GEDT values (Table 3). The further conversion of **TS**s directly led to the valleys associated with the existence of corresponding products. All attempts to optimize the postulated zwitterionic intermediates were not successful. The detailed analysis of the IRC trajectories showed, however, that both considered reactions should be defined as “*two-stage one-step*” processes [42]. The mechanism discussed above was subsequently evaluated on the basis of quantum-chemical calculations using more advanced levels of theory. In all cases, very similar results were obtained. This fact confirms that the B3LYP/6-31G(d) level of theory is fully adequate for resolving the title issue, and the application of higher levels of theory is completely unnecessary.

In the next step, similar studies were performed for the same reactions in the simulated presence of different types of solvents. It was found that, both in low-polar chloroform and very polar ethanol, the considered reaction channels took place via a one-step mechanism through the formation of pre-reaction molecular complexes. All attempts to optimize the theoretically possible zwitterionic intermediates were not successful. The quantitative descriptions of the reaction profiles, as well as the critical structures, were, however, slightly different from those performed in the gas phase. In particular, valleys associated with the existence of **MC**s were slightly shallower. At the same time, activation enthalpies in the solution were higher than in the gas phase, though not to the extent that any of the reactions could be considered kinetically forbidden. A certain increase in the activation enthalpy was accompanied by an increase in the asynchronicity of the transition states. However, this effect was not pronounced enough to force the reaction to proceed through a two-step mechanism with a zwitterionic intermediate.

The observed solvent effects are in agreement with the available information in the literature regarding the influence of the polarity of solvents on the course of 32CAs. In particular, it is well known that in the case of one-step, polar 32CAs, the replacement of low polar solvents by more polar solvents can determine the multiple changes of rate constants [43,44]. On the other hand, in stepwise cycloadditions, the replacement of low-polar solvents by more polar solvents can determine the 1000-fold and greater change of rate constants [45].

## 3. Computational Procedure

All calculations reported in this paper were performed on a Prometheus computer cluster in the CYFRONET regional computational center in Cracow. Hybrid functional B3LYP with the 6-31G(d) basis set included in the GAUSSIAN package [46] was used. Though the B3LYP/6-31G(d) level of theory is quite simple, it should be emphasized that it is still perfectly suited for modeling the mechanisms of organic reactions, especially cycloaddition reactions. Recently, experimental and theoretical determinations of activation enthalpies and kinetic isotope effects (directly correlated with the degree of rehybridization, and thus the degree of advancement of new bonds) for different types of cycloaddition reactions have been performed. In these works, a perfect agreement was obtained between the experimentally measured parameters and those calculated with the use of the B3LYP functional [47,48,49]. Subsequently, for the model reaction, analogous calculations were performed using more advanced levels of theory. In all cases, results close to the B3LYP/6-31G(d) data were obtained. Therefore, the issues mentioned above confirm, without any doubts, that the B3LYP/6-31G(d) level of theory is fully adequate for resolving the problem defined in the Introduction.

Stationary points were characterized by frequency calculations. All reactants and products had positive Hessian matrices. All transition states showed only one negative eigenvalue in their diagonalized Hessian matrices, and their associated eigenvectors were confirmed to correspond to the motion along the reaction coordinate under consideration. For all reactions, IRC calculations were performed to connect the previously computed transition structures with suitable minima. For the calculations of solvent effects on the reaction paths, the polarizable continuum model (PCM) [50,51] was applied. Values of the global electron density transfer (GEDT) [52] were calculated according to the formula:

GEDT = Σq_A_ (1)

where q_A_ is the net charge, and the sum is taken over all the atoms of nitrone.

Global electronic properties of reactants were estimated according to the equations recommended earlier by *Parr* and *Domingo* [34]. In particular, the electronic chemical potentials (μ) and chemical hardness (η) were evaluated in terms of one-electron energies of FMO (Ε_HOMO_ and Ε_LUMO_) using the following equations:

μ ≈ (E_HOMO_ + E_LUMO_) / 2GEDT = Σq_A_ (2)

η ≈ E_LUMO_ − E_HOMO_ (3)

Next, the values of ω and η were then used for the calculation of the global electrophilicity (ω) according to the formula:

ω = μ^2^/2η, (4)

Subsequently, the global nucleophilicity (N) [53] can be expressed in terms of the equation:

N = E_HOMO_ − E_HOMO (tetracyanoethene)_ (5)

The local electrophilicity (ω_k_), condensed to atom k, was calculated by projecting the index ω onto any reaction center k in the molecule by using Parr functions P^+^_k_ [54]:

ω_k_ = P^+^_k_ × ω (6)

The local nucleophilicity (*N_k_*), condensed to atom *k,* was calculated using the global nucleophilicity *N* and *Parr* functions P^−^_k_ [54] according to the formula:

*N*_k_ = P^−^_k_ × *N* (7)

All calculated reactivity indices are collected in Table 1.

## 4. Conclusions

The latest discoveries in the field of mechanistic aspects of [3 + 2] cycloaddition (32CA) reactions refute the once-fashionable thesis that one-step mechanisms are the only possible ones, regardless of the nature of the reactants. At this moment, some cases of stepwise, zwitterionic mechanisms regarding 32Cas, involving different types of three-atom components, are known. A similar mechanism is theoretically possible in the case of the 32CA between C-arylnitrones and perfluoro 2-methylpent-2-ene. This assumption can be supported by the strong electrophilic nature of the alkene system, the presence of fluorine atoms near the reaction centers, and the presence of acyclic adducts in the postreaction mixture. The DFT computational study shows, however, that all considered cycloadditions proceed via a one-step mechanism. All attempts for the localization of zwitterionic intermediates on the reaction paths were not successful. The detailed analysis of IRC trajectories shows that, according to Domingo’s terminology, the considered reactions should be defined as “*two-stage one-step*” processes.

## Figures and Tables

**Table 1:** Global and local electronic properties of nitrones **1a**–**c** and perfluoro 2-methylpent-2-ene **2** expressed in eV.

	Global Properties		Local Properties			
	ω	N	N_C2/C_	N_C3/O_	ω_C2/C_	ω_C3/O_
**1a**	1.33	3.88	0.01	2.14	0.04	0.30
**1b**	1.57	3.51	0.28	2.11	0.01	0.36
**1c**	1.61	3.46	0.23	2.05	0.03	0.37
**2**	2.38	0.15	0.08	0.04	0.68	1.12

**Table 2:** DFT kinetic and thermodynamic parameters for the 32CAs of nitrone **1b** with perfluoro 2-methylpent-2-ene **2** (ΔH, ΔG are in kcal/mol; ΔS are in cal/molK).

Environment	Path	Transition	ΔH	ΔG	ΔS
Gas phase		**1b + 2→MCA**	−6.3	6.9	−44.3
	**A**	**1b + 2→TSA**	2.8	18.7	−53.1
		**1b + 2→3b**	−27.9	−10.4	−58.5
		**1b + 2→MCB**	−5.5	5.5	−37.0
	**B**	**1b + 2→TSB**	1.9	17.8	−53.3
		**1b + 2→4b**	−24.3	−6.5	−59.7
Chloroform		**1b + 2→MCA**	−3.9	7.6	−38.3
	**A**	**1b + 2→TSA**	3.3	18.4	−50.5
		**1b + 2→3b**	−24.9	−8.0	−56.7
		**1b + 2→MCB**	−3.8	7.9	−39.4
	**B**	**1b + 2→TSB**	2.4	18.1	−52.6
		**1b + 2→4b**	−21.2	−3.8	−58.1
Ethanol		**1b + 2→MCA**	−3.3	8.0	−37.8
	**A**	**1b + 2→TSA**	3.3	18.1	−49.7
		**1b + 2→3b**	−23.8	−7.0	−56.6
		**1b + 2→MCB**	−3.3	7.8	−37.4
	**B**	**1b + 2→TSB**	2.4	18.2	−52.7
		**1b + 2→4b**	−20.1	−2.7	−58.2

**Table 3:** Selected parameters of key structures of the 32CAs of nitrone **1b**, with perfluoro 2-methylpent-2-ene **2** obtained from DFT calculations.

Environment	Structure	Interatomic Distances (Å)					I_C3-C4_	I_C5-O1_	ΔI	GEDT (e)
		O1-N2	N2-C3	C3-C4	C4-C5	C5-O1				
Gas phase	**MCA**	1.275	1.310	3.827	1.341	2.748	0.435	0.686	0.25	0.00
	**TSA**	1.316	1.324	2.511	1.413	1.816				0.19
	**3b**	1.444	1.472	1.604	1.583	1.383				
	**MCB**	1.276	1.310	3.822	1.341	2.676	0.418	0.747	0.33	0.00
	**TSB**	1.318	1.325	2.532	1.420	1.764				0.19
	**4b**	1.434	1.471	1.600	1.586	1.408				
Chloroform	**MCA**	1.281	1.309	3.741	1.340	2.759	0.371	0.733	0.36	0.00
	**TSA**	1.326	1.318	2.616	1.417	1.752				0.26
	**3b**	1.446	1.473	1.606	1.582	1.382				
	**MCB**	1.282	1.308	3.698	1.340	2.678	0.395	0.799	0.40	0.00
	**TSB**	1.330	1.322	2.571	1.429	1.691				0.25
	**4b**	1.436	1.472	1.601	1.585	1.408				
Ethanol	**MCA**	1.284	1.308	3.737	1.340	2.783	0.332	0.751	0.42	0.00
	**TSA**	1.330	1.315	2.679	1.418	1.727				0.30
	**3b**	1.447	1.473	1.606	1.582	1.383				
	**MCB**	1.285	1.307	3.737	1.340	2.702	0.391	0.822	0.43	0.00
	**TSB**	1.335	1.322	2.577	1.435	1.658				0.28
	**4b**	1.436	1.472	1.602	1.585	1.408				

## Data Availability

Not applicable.
